# High Pressure Synthesis of Pr_2_O_5_ – A Unique Lanthanoid(IV) Oxide Peroxide

**DOI:** 10.1002/anie.202422929

**Published:** 2025-02-13

**Authors:** Niko T. Flosbach, Lukas Brüning, Pascal L. Jurzick, Elena Bykova, Marcus Ekholm, Igor A. Abrikosov, Mohammad Amirabbasi, Mohamed Mezouar, Björn Wehinger, Nico Giordano, Timofey Fedotenko, Vitali Prakapenka, Stella Chariton, Mathias S. Wickleder, Maxim Bykov

**Affiliations:** ^1^ University of Cologne Institute of Inorganic Chemistry Greinstr. 6, 50939 Cologne Germany; ^2^ Goethe University Frankfurt Institute of Inorganic and Analytical Chemistry Max-von-Laue-Str. 7, 60438 Frankfurt am Main Germany; ^3^ Goethe University Frankfurt Institute of Geosciences Altenhöferallee 1, 60438 Frankfurt Germany; ^4^ Linköping University Department of Physics Chemistry and Biology SE-581 83 Sweden; ^5^ Fachgebiet Materialmodellierung Institut für Materialwissenschaft Technische Universität Darmstadt Otto-Berndt-Str. 3, D-64287 Darmstadt Germany; ^6^ European Synchrotron Radiation Facility Grenoble Cedex F-38043 France; ^7^ Deutsches Elektronen Synchrotron 22607 Hamburg Germany; ^8^ University of Chicago Center for Advanced Radiation Sources Chicago, IL USA

**Keywords:** High-pressure, lanthanides, oxidation, praseodymium, X-ray diffraction

## Abstract

Reacting praseodymium(IV) oxide with oxygen at 27 GPa in a diamond anvil cell yielded the oxide peroxide Pr_2_^IV^(O_2_)O_3_, which was characterized by single crystal X‐ray diffraction on multi‐grain samples, Raman spectroscopy and quantum theoretical calculations at various pressure points. The presence of tetravalent praseodymium ions is supported by electronic structure calculations, showing a band gap of ca. 1.2 eV, which is consistent with the anticipated chemical model of an ionic solid. Pr_2_(O_2_)O_3_ thus far represents the most oxygen rich phase of any binary compound of a lanthanoid and oxygen and is the first example of a peroxide anion next to Pr^4+^. Additionally, these results demonstrate that instead of oxidizing the praseodymium ions past their +IV oxidation state, oxygen undergoes a comproportionation to form peroxide anions. Direct oxidation of the oxide anions by Pr^4+^‐ions was ruled out by a control experiment in argon instead of oxygen, where no oxidation of oxide ions was observed.

## Graphical abstract

The reaction of PrO_2_ and oxygen at 27 GPa in a diamond anvil cell afforded the unique oxide peroxide Pr_2_(O_2_)O_3_ in which praseodymium is found in its rare oxidation state of +IV. Pr_2_(O_2_)O_3_ constitutes the most oxygen rich binary phase of any lanthanoid and oxygen. It could be characterised by X‐ray single crystal diffraction analysis at various pressure points, Raman spectroscopy and theoretical calculations.

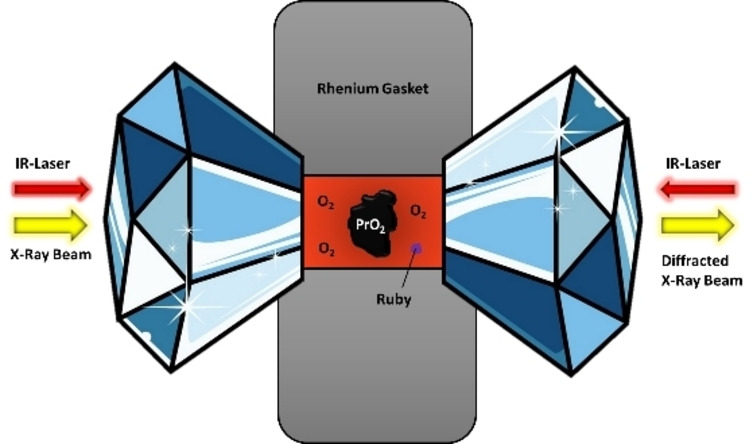

The unique physical and chemical properties of the lanthanoids make them essential components in modern technologies, including electronics, optical devices, and magnetic materials.^[1]^ The chemistry of the lanthanoids is dominated by the +III oxidation state that is the most stable for all elements of the series. However, variations in the electronic configuration of lanthanoids in different oxidation states significantly influence the physical properties of their ions. Consequently, the search for new compounds containing lanthanoids in oxidation states other than +III is not only important for the expanding the fundamental knowledge of their chemistry but is also motivated by the potential of developing novel materials of unprecedented physical and chemical properties.

Some elements of the series can form stable compounds in their divalent oxidation states. The *classical* divalent lanthanoids are samarium, europium and ytterbium. Due to their chemical availability, research on divalent lanthanoids has historically been concentrated on these three elements, albeit recent studies have shown that the entire series, except for radioactive promethium, is accessible in their divalent oxidation state, at least in metal organic complexes.[^2^, ^3^, ^4^] Additionally, several *non‐classical* lanthanoids can be found in the form of reduced halides.^[5]^

In contrast to the divalent lanthanoids, the accessibility of their tetravalent counterparts is much more difficult because of the strongly oxidizing character of the respective ions. In fact, the Ce^4+^‐ion is the only Ln^4+^‐ion that is available in a wide variety of compounds, albeit it is still strongly oxidizing. When comparing the standard oxidation potentials E_0_ for the Ln^3+^/Ln^4+^ pairs, Pr^3+^/Pr^4+^ (E_0_=+3.2 V) and Tb^3+^/Tb^4+^ (E_0_=+3.1 V) follow Ce^4+^ (E_0_=+1.74 V) in the row of the most stable Ln^4+^‐ions.[^6^, ^7^] As expected for such strongly oxidizing cations, the chemistry of Pr^4+^ and Tb^4+^ is almost exclusively limited to oxides and fluorides.[^8^, ^9^, ^10^, ^11^, ^12^, ^13^, ^14^, ^15^, ^16^, ^17^, ^18^, ^19^, ^20^, ^21^, ^22^, ^23^, ^24^] Even simple salts of the form LnO_2_ or LnF_4_ (for Ln=Pr, Tb) usually require harsh conditions to be synthesized.^[25]^ The recent discovery of metal organic complexes containing Pr^4+^ and Tb^4+^, as well as the synthesis of K_2_Tb^IV^Ge_2_O_7_ and the mixed valent Cs_8_Tb_2_^III^Tb^IV^Ge_9_O_27_ present an evidence that the chemistry of tetravalent praseodymium and terbium can be expanded beyond simple oxides and fluorides, thus providing the opportunity to investigate their optical and magnetic behavior in more detail.[^26^, ^27^, ^28^, ^29^, ^30^, ^31^, ^32^, ^33^, ^34^, ^35^, ^36^] Combined theoretical and experimental studies of selected examples of Pr^4+^ containing compounds have shown that the 4f^1^‐configured ion does not follow the general trends typically observed in 4f^n^‐configured ions but rather behaves more similar to the early actinoids.^[37]^ Given the scarcity of materials containing Pr^4+^, such studies thus far have to remain focused on very few systems, limiting the opportunity to establish new trends and to research potential applications.

Although Nd^4+^ and Dy^4+^ show an even higher proclivity to be reduced to their respective trivalent ions (E_0_=+5.0 V for Nd^3+^/Nd^4+^ and E_0_=+5.2 V for Dy^3+^/Dy^4+^),[^6^, ^7^] fluorides of the form Cs_2_MLn^IV^F_7_ (M=K, Rb, Cs; Ln=Nd, Dy) have been prepared by oxidation with fluorine under extreme pressure.[^38^, ^39^] The binary fluorides Ln^IV^F_4_ (Ln=Nd, Dy) have thus far only been observed in noble gas matrices.^[40]^ When attempting to synthesize a perovskite of the form BaNd^IV^O_3_ by reacting barium peroxide with neodymium(III) oxide at 4 GPa and 1500 °C Range et al. obtained an oxide peroxide with the empirical formula NdO_2_, which is to be conceived as Nd_2_^III^(O_2_)O_2_, as evidenced by single crystal structure analysis and vibrational spectra.[^41^, ^42^, ^43^] This unique discovery poses the questions if oxide peroxides also exist with lanthanoids in higher oxidation states than +III if the synthetic procedure is modified accordingly.

Recent studies have revealed that a multitude of unique compounds like CaO_3_, Xe_3_O_2_, Xe_2_O_5_ or FeO_2_, which are inaccessible by other methods, can be synthesized by utilizing the reactivity of oxygen at high pressure.[^44^, ^45^, ^46^]

We herein report the synthesis and crystal structure of the unique oxide peroxide Pr_2_^IV^(O_2_)O_3_ with the empirical formula Pr_2_O_5_ that was obtained by oxidation of PrO_2_ at 27 GPa in a diamond anvil cell (Scheme 1).

**Scheme 1:**
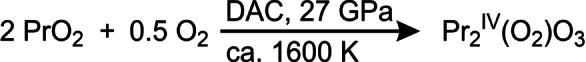
The oxidation of PrO_2_ with O_2_ at 27 GPa in a diamond anvil cell (DAC) resulted in the formation of Pr_2_(O_2_)O_3_.

The title compound could be identified by single crystal X‐ray diffraction of multi grain samples at three different pressure points (27 GPa, 20 GPa and 11 GPa, see Table S1). The crystal structure is discussed in more detail below. In situ Raman spectroscopy of the reaction product in a DAC revealed two weak signals at 820 cm^−1^ and 848 cm^−1^ that are assigned to characteristic vibrations of the peroxide anion based on comparison of similar compounds (Figure 1).[^47^, ^48^]

**Figure 1:**
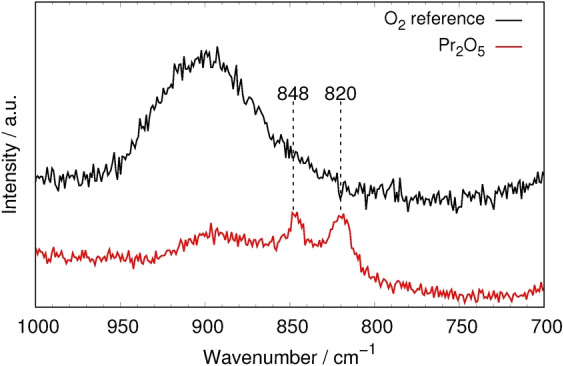
Excerpt of the Raman spectrum of the reaction chamber at 27 GPa. Vibrations assigned to the peroxide group of Pr_2_(O_2_)O_3_ are highlighted. The spectra were recorded with an excitation wavelength of 532 nm. The black spectrum is a reference spectrum of only oxygen without praseodymium oxides that was measured using the same experimental setup.

The weak signal to noise ratio is caused by the small quantities of Pr_2_(O_2_)O_3_ next to other materials as a result of local heating. In order to exclude autooxidation of praseodymium dioxide as a possible reaction pathway to Pr_2_(O_2_)O_3_, the experiment was repeated using argon as the pressure transmitting medium instead of oxygen, where the cotunnite‐type PrO_2_ was observed as the main phase, proving that a second reactant is needed for the formation of Pr_2_(O_2_)O_3._ ^[49]^

Because the general structure motifs are the same for all three pressure points (27, 20 and 11 GPa), the details are discussed for the structure at 11 GPa which is the closest to ambient pressure. Pr_2_(O_2_)O_3_ crystallizes in the orthorhombic space group *Pbcn* (no. 60) with four formula units per unit cell. The Pr^4+^‐ions form a distorted body‐centered cubic (*bcc*) sublattice in which the oxide anions O2 and O3 occupy tetrahedral voids and the peroxide ions occupy octahedral voids (Figure 2, Figure 3).

**Figure 2:**
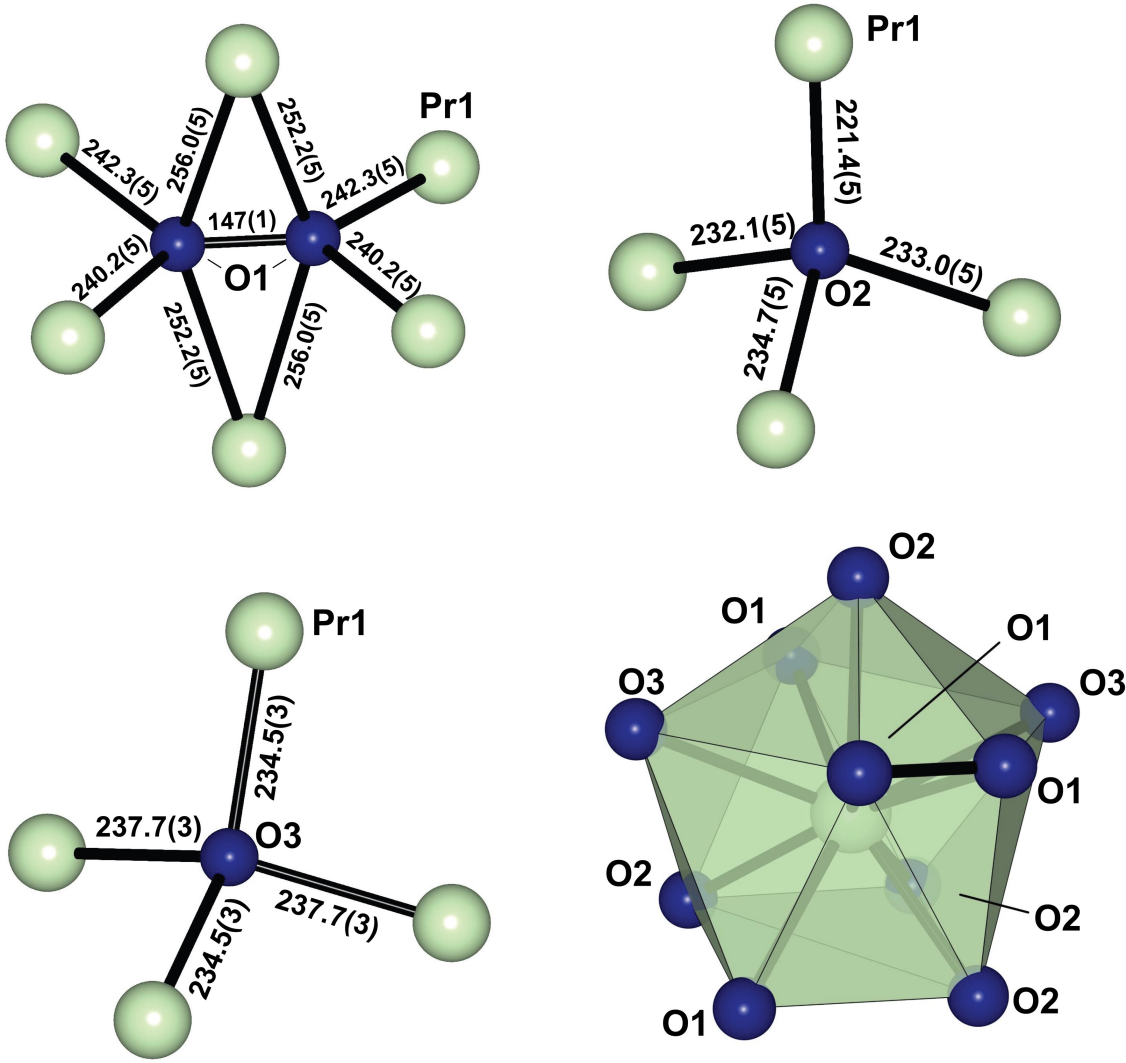
Coordination geometries of the peroxide anion, the oxide anions O2 and O3 and the Pr^4+^‐ion. The displayed interatomic distances refer to the structure at 11 GPa. Distances are given in pm.

**Figure 3:**
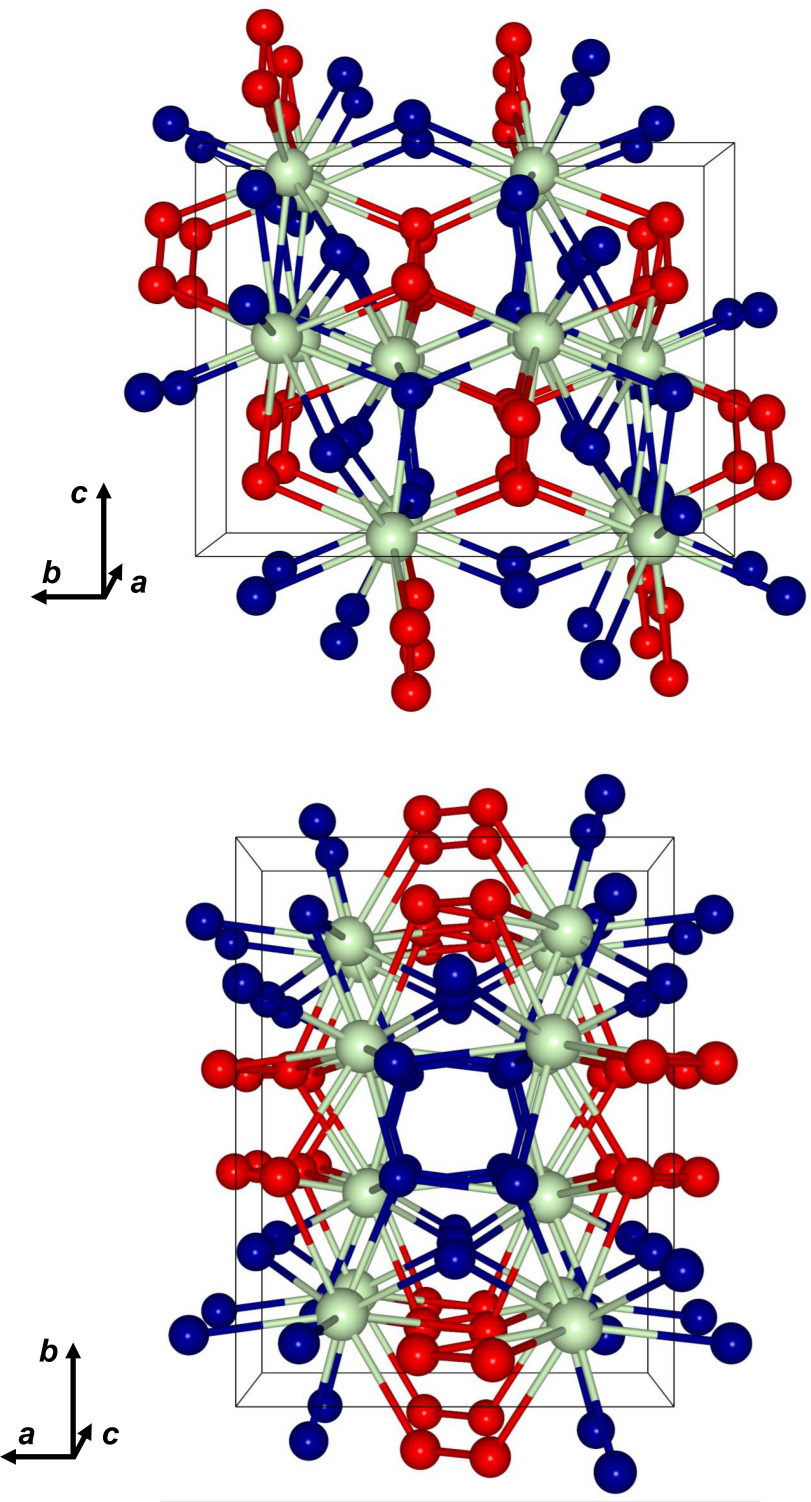
Crystal structure of Pr_2_(O_2_)O_3_ at 11 GPa viewed along the crystallographic *a*‐ and *c*‐axes. Green=praseodymium; blue=oxide; red=peroxide.

The [OPr_4_]‐polyhedra of O2 and O3 deviate significantly from the ideal tetrahedral geometry. In the coordination polyhedron of O2 the Pr1‐O2‐Pr1 angles reach as high as 123.8(3)° and as low as 99.6(2)° with an average angle of 109.4°. The Pr−O2 distances range from 234.7(5) pm to 221.4(5) pm with an average value of 230.3 pm across all four bonds. The Pr‐O3 distances are slightly longer. Because O3 lies on a twofold rotational axis (Wyckoff Symbol=*4c*), there are only two different Pr−O distances to be discussed (234.5(3) pm and 237.7(3) pm). The deviation of the [OPr_4_]‐polyhedron around O3 from ideal tetrahedral geometry is even more pronounced than in the polyhedron of O2. The largest Pr1‐O3‐Pr1 angle found at 11 GPa is 137.3(3)°, which is much larger than the ideal tetrahedral angle of ca. 109.5°. On the contrary, the smallest angle in the same polyhedron is only 97.73(1)°, further indicating a strong distortion when compared to ideal tetrahedral geometry. This distortion is a result of the significant deviation of the sublattice of Pr^4+^‐ions from the ideal body centered cubic packing. The peroxide anions constituted of O1 atoms occupy octahedral voids in the *bcc* sublattice of Pr^4+^‐ions, as illustrated in Figure 2. On two opposing sides the peroxide anions coordinate to one Pr1 site with both atoms (*η* ^*2*^) each, with Pr−O distances 256.0(5) pm and 252.3(5) pm. The terminal coordination to Pr1 includes four Pr^4+^‐ions, two for each oxygen atom, where the distances are found to be 242.3(5) pm and 240.2(5) pm. With an O‐O distance of 146(1) pm, the O−O‐bond lies within the distance typically observed for peroxide anions, even when high pressure is accounted for.[^47^, ^48^, ^50^]

The coordination polyhedron of Pr1 involves ten oxygen atoms, wherein four are constituents of peroxide anions while the remaining six are constituents of oxide anions. The coordination polyhedron of the Pr^4+^‐ions can be labeled as a distorted augmented sphenocorona (*Johnson*‐solid no. 87) with a distortion coefficient of 2.427, as suggested by CShM‐calculations.^[51]^ The average Pr−O distance in the polyhedron is 238.4(5) pm, where the shortest distance is found between Pr1 and O2 at 221.4(5) pm and the longest between the *η* ^*2*^‐coordinating peroxide ion and Pr1 (256.0(5) pm). Despite the higher coordination number of the Pr^4+^‐ion, compared to the 8‐fold coordination of Nd^3+^ in Nd_2_^III^(O_2_)O_2_ (d_avg_(Nd‐O)=247 pm), the distances between lanthanoid ion and oxygen atoms are significantly shorter, suggesting a higher charge of the cation, even when considering the higher pressure.^[41]^ The ionic radius of Pr^3+^ is approximately 118 pm for the case of 9‐fold coordination (no value is available for 10‐fold coordination) and that of a 4‐fold coordinated oxide anion 138 pm,^[52]^ yielding an expected Pr^3+^−O^2−^‐distance of 256 pm, which is much longer than that observed in the title compound (Figure 2).

At higher pressures Pr_2_(O_2_)O_3_ still crystallizes in the same orthorhombic space group *Pbcn*, with the same structure that was found at 11 GPa. A comprehensive overview of systematic trends of the pressure dependence of selected structural motifs is provided in the following abstract.

The most fundamental change at higher pressure is the compression of the entire structure, resulting in shortening of the lattice parameters and hence the molar volume of its contents. The lattice parameters are plotted for all experimental pressure points along with theoretically calculated data in Figure 4. The cell volume decreases from 305.33(7)· 10^6^ pm^3^ to 286.19(1)· 10^6^ pm from 11 GPa to 27 GPa by ca. 6.3 %.

**Figure 4:**
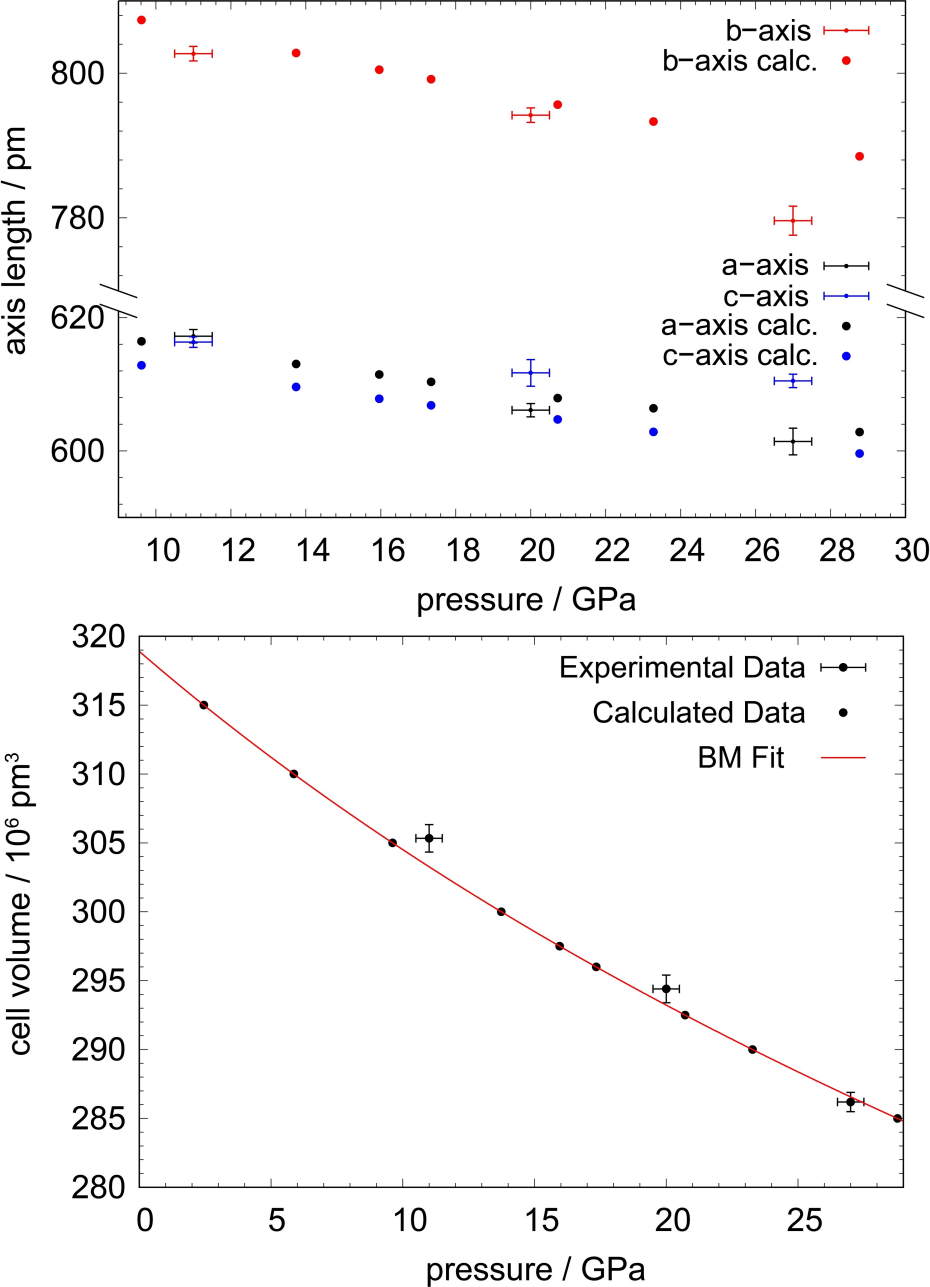
Lattice parameters of Pr_2_(O_2_)O_3_ at different pressure points (top) and cell volume of the unit cell at different pressures (bottom). Calculated values are displayed at points, whereas experimental data is shown as crosses. The red line in the bottom graphic symbolizes the Birch‐Murnaghan equation of state obtained by theoretical calculations.

While the *c*‐axis length only decreases from 616.33(8) pm at 11 GPa to 610.5(5) pm at 27 GPa, which is equivalent to a relative compression of ca. 1.0 %, the *a*‐ and *b*‐axes are more strongly compressed. The *a*‐axis length decreases from 617.2(1) pm to 601.4(2) pm, corresponding to a relative change of ca. 2.5 %, whereas the *b*‐axis undergoes a slightly larger relative change of 2.9 % from 802.7(1) pm to 779.6(2) pm. The decline of axis length along the *a*‐ and *b*‐axes does not follow a linear trend, whereby the compression of the *c*‐axis is linear within the margin of error. Naturally, the compression of the cell is facilitated by shortening of chemical bonds. The O‐O distance of the peroxide group is shortened by ca. 2 % from 147(1) pm at 11 GPa to 144.1(7) pm at 27 GPa, as is depicted in figure 5.

**Figure 5:**
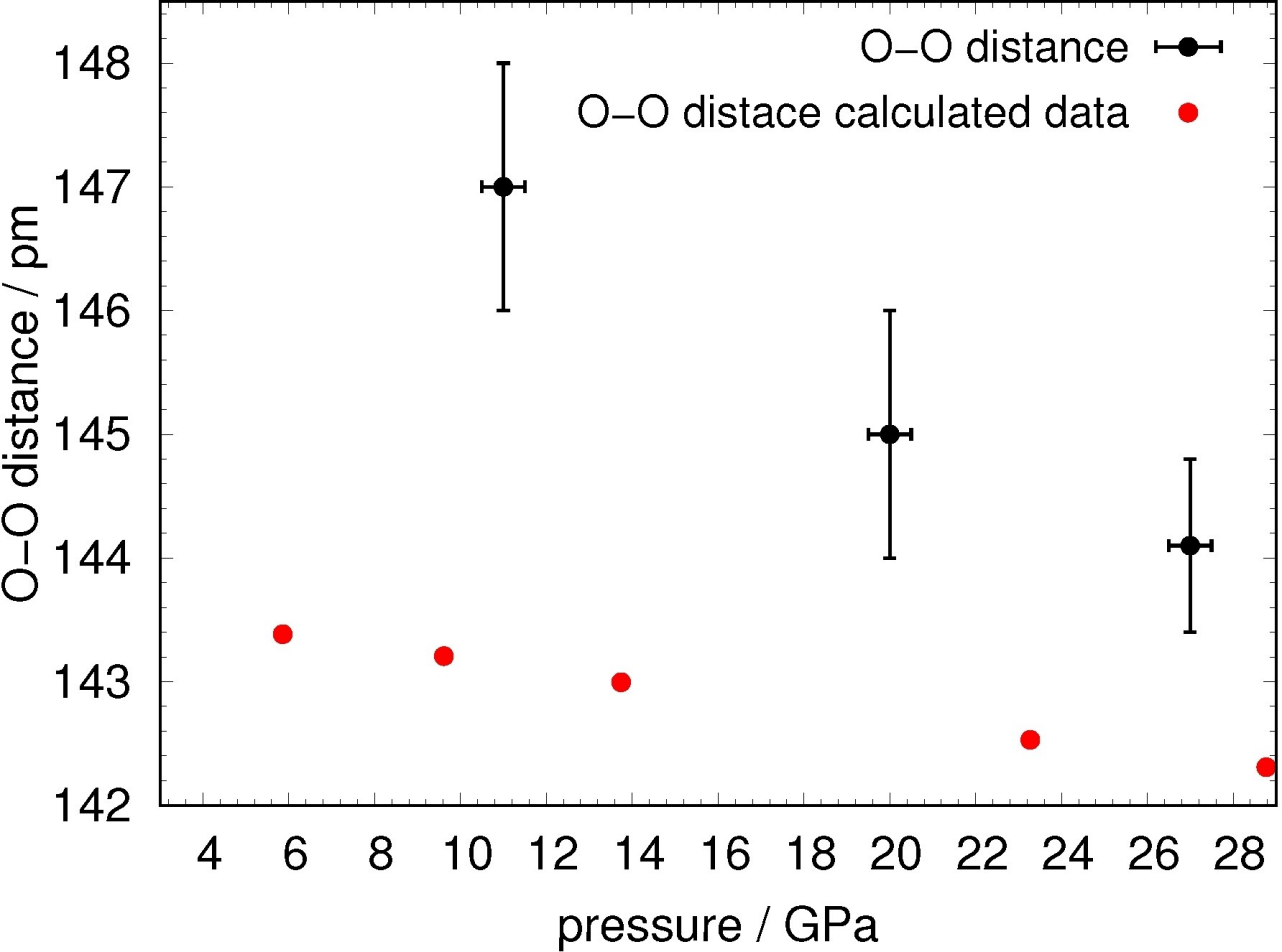
Pressure dependence of the O−O distance in the peroxide anion in Pr_2_(O_2_)O_3_. Calculated data is shown in red.

With increasing pressure, the [PrO_10_]‐polyhedron loses approximately 6.2 % of its volume between 11 to 27 GPa (285.1(2)· 10^6^ pm^3^ to 267.3(2)· 10^6^ pm^3^). Likewise, the average Pr−O distance in the polyhedron decreases from 238.4 pm at 11 GPa to 233.3 pm at 27 GPa. All discussed distances are shown in tab. S2.

The ab‐initio calculations recover the orthorhombic *Pbcn* structure at all volumes. We also obtain good quantitative agreement with the experimental lattice parameters. Nevertheless, the monotonic pressure dependence of the theoretically predicted lattice parameters deviate slightly from the experimentally observed trends, which is likely due to the non‐hydrostatic conditions in the cell. By fitting the the Birch‐Murnaghan equation of state to the calculated pressure‐volume data we obtain the bulk modulus B_0_=192.8 GPa and B_0_’=5.0, with the equilibrium volume V_0_=319.2· 10^6^ pm^3^.^[53]^ The calculations also reproduce a decreased peroxide distance with pressure, as seen in figure 5, in support of the experiments.

The electronic structure calculations (see Figure S5) reveal an insulating band gap of 1.2 eV and a local Pr magnetic moment of ~1 μ_B_. This is consistent with the model of an ionic salt containing 4f^1^‐configured Pr^4+^ ions, along with oxide and peroxide anions each carrying localized negative charges. We suggest that the formation of the +IV oxidation state is possible due to the peroxide dimers, which form antibonding states and prevent oxidation of Pr beyond +IV. This is supported by the calculated electronic density of states which shows the antibonding states of the peroxide anions, accommodating two electrons per formula unit (see SI).

The reaction of praseodymium dioxide with oxygen at 27 GPa afforded the unprecedented lanthanoid(IV) oxide peroxide Pr_2_^IV^(O_2_)O_3_ with the empirical formula Pr_2_O_5_ with the highest oxygen content of any binary phase of any lanthanoid and oxygen. Pr_2_(O_2_)O_3_ is only the second oxide peroxide to be discovered, adding another member to this exclusive group of compounds. Its structure was determined by single crystal X‐ray diffraction techniques and further corroborated by quantum theoretical calculation as well as Raman spectroscopy. Upon decompression of the sample, the title compound decomposed into unknown products between 11 GPa and ambient pressure. Investigation of the compound at different pressure points between 11 GPa and 27 GPa, along with theoretical calculations allowed for the determination of the Birch‐Murnaghan equation of state of Pr_2_(O_2_)O_3_.

In our experiments, no evidence of oxidation of praseodymium past the +IV oxidation state was observed, indicating that the +V oxidation state is not accessible under the applied experimental parameters, at least not in binary oxides. The presence of tetravalent praseodymium ions next to peroxide anions has not been reported before and to the best of our knowledge has never been reported for any other tetravalent lanthanoid cation. The electronic structure was assessed by band structure calculations that support the assignment of charge distribution within the solid. These discoveries set the foundation for further studies of the physical and chemical properties of the 4f^1^‐configured Pr^4+^‐ion that have recently been demonstrated to deviate from the general trends observed in lanthanoids.^[37]^

## Supporting Information

Experimental Details; Crystallographic data of Pr_2_(O_2_)O_3_ (Table S 1); Summary of selected interatomic distances (Table S 2); Full Raman spectrum (Figure S 1), XRD‐map at 27 GPa (Figure S 2); XRD‐map at 20 GPa (fig. S 3); XRD‐map at 11 GPa (Figure S 4); Calc. density of states plots (Figure S 5). Deposition Numbers 2303283 (for structure at 27 GPa), 2303282 (for structure at 20 GPa) and 2303284 (for structure at 11 GPa) contain the supplementary crystallographic data for this paper. These data are provided free of charge by the joint Cambridge Crystallographic Data Centre and Fachinformationszentrum Karlsruhe http://www.ccdc.cam.ac.uk/structures.

## Conflict of Interests

The authors declare no conflict of interest.

## Supporting information

As a service to our authors and readers, this journal provides supporting information supplied by the authors. Such materials are peer reviewed and may be re‐organized for online delivery, but are not copy‐edited or typeset. Technical support issues arising from supporting information (other than missing files) should be addressed to the authors.

Supporting Information

## Data Availability

The data that support the findings of this study are available in the supplementary material of this article. Data collected at the ESRF are availabhe at doi.org/10.15151/ESRF‐DC‐2044641962
